# The Mediating Effect of Coping Strategies Between Psychological Capital and Small Tourism Organization Resilience: Insights From the COVID-19 Pandemic, Malaysia

**DOI:** 10.3389/fpsyg.2021.766528

**Published:** 2021-12-03

**Authors:** Muhammad Farhan Jalil, Azlan Ali, Zeeshan Ahmed, Rashidah Kamarulzaman

**Affiliations:** ^1^School of Business and Management, University College of Technology Sarawak, Sibu, Malaysia; ^2^Department of Business Management, University of Lahore Gujrat Campus, Gujrat, Pakistan

**Keywords:** psychological capital, coping strategies, problem-focused, emotion-focused, organizational resilience, small tourism firms

## Abstract

Amid difficulty, the psychological capital of small tourism firm owners/managers has been given less attention. In the coronavirus disease-2019 (COVID-19) pandemic, this research examined how psychological capital (self-efficacy, hope, optimism, and resilience) affects organizational resilience. By structural equation modeling (AMOS 21.0), 644 small tourism firm owners in Malaysia were randomly selected to investigate the relationship between psychological capital and organizational resilience, and the mediating effect of problem-focused and emotion-focused coping strategies on this relationship. The findings of the study supported hypothesized relationships, as the psychological capital of small tourism firm owners in Malaysia significantly affects organizational resilience. Furthermore, the study discovered that problem-focused and emotion-focused coping strategies have partial mediating effects on the association between psychological capital and organizational resilience. In the context of small tourism businesses sector, the findings of the study have implications, as the firms identify the recovery procedure in the COVID-19 pandemic.

## Introduction

With rise in frequency and effects, coronavirus disease-2019 (COVID-19) has become more common all over the world (Popkin et al., 2020; Mishra et al., 2021). Recent evidence indicates that a new trend is emerging with COVID-19, with decreased mortality but increased costs (Cherry et al., 2020). Since epidemics such as COVID-19 influence tourism supply and demand simultaneously, the tourism sector is extremely sensitive to natural disasters (González-Torres et al., 2021). In the same way, Škare et al. (2021) have discussed huge consequences on tourism following the massive COVID-19 pandemic that hit the world. Moreover, the effects of the COVID-19 pandemic on the tourism sector were “rapid, significant, and persistent.”

Many epidemics, such as severe acute respiratory syndrome (SARS) in 2003, influenza A (H1N1) in 2009, Middle East respiratory syndrome (MERS) in 2012, and Ebola in 2014 (Zeng et al., 2005; Lee et al., 2012; Novelli et al., 2018), have seriously affected the tourism industry during the last 2 decades, as travel might aggravate health problems and it is best to avoid it. In the tourism sector, there has been several studies on health problems and tourism disaster management (Pforr and Hosie, 2008; Jamal and Budke, 2020). The major concern of previous studies has been on the flow of tourists and revenue generation due to the influence of epidemics. However, fewer studies have been undertaken on how owners/managers of small tourism firms can develop the psychological capital to help their businesses recover quickly during or after crisis (Pathak and Joshi, 2020). According to Prayag et al. (2020), understanding the impact of psychological capital and organizational resilience has become critical, since both concepts have unique significance for organizations undergoing any type of restructuring. Moreover, Luthans (2002) described that the development and sustainability of an organization are largely dependent on owners/managers in this period of uncertainty. Hence, the aim of this study is to examine the impact of the psychological capital of small tourism firm owners/managers on organizational resilience during the COVID-19 pandemic. According to Luthans (2002), psychological capital is defined as “an individual’s positive psychological condition of development” as shown by four components: self-efficacy, hope, optimism, and resiliency. Pathak and Joshi (2020) described organizational resilience as “organization’s perceived capacity to overcome disruptions and accept change.”

The literature on disaster management emphasizes the difficulties that individuals and businesses confront in the aftermath of a disaster. Businesses must find new strategies to adapt to the changing climate (Andersson and Keskitalo, 2018). According to Lazarus and Folkman (1984), a coping strategy is an activity, a sequence of acts, or a mental process that is used to deal with a stressful or unpleasant circumstance or to change the attitude of one to it. In contrast to defensive systems, coping methods usually entail a deliberate and direct approach to difficulties (Abbas et al., 2020). The importance of psychological capital in coping strategies and recovery from tough situations has been highlighted in the research. In terms of coping strategies in a pandemic, the present literature supports the “individual” viewpoint above “business.” One major gap in the past studies is in what way coping strategies help the development of resiliency in small tourism firms.

The importance of psychological capital in small businesses is often crucial to the recovery of a business, and it may affect the resilience of the business (Mao et al., 2020; Prayag et al., 2020). However, it is unclear how psychological capital shows itself and how it affects organizational resilience. In the tourism industry, Pathak and Joshi (2020) pointed out that little attention has been paid to establish or sustain organizational resilience in crisis situations. More crucially, little emphasis has been paid to the influence of psychological capital on the resilience of small tourism businesses in the literature. Individuals are said to use coping strategies (Schroder et al., 2017; Baloran, 2020), but it has an impact on organizational resilience, and so far it has not yet been studied among small firms in pandemics. Given that coping strategies are positively associated with psychological capital, individuals must be able to deal with and adapt to the change in order to be persistent (Rabenu et al., 2017). However, studies on the association among psychological capital, coping strategies, and organizational resilience are actually needed.

Therefore, this study adds to the literature by including psychological capital into the resilience of small tourism businesses during pandemics, and the novelty of the study is to identify the mediating impact of coping strategies between the relationships. Current research on the resiliency of small tourism businesses (Prayag et al., 2020; Williams et al., 2020; Sobaih et al., 2021) has not adequately established how owners/managers of small business use psychological capital to promote organizational resilience in the COVID-19 pandemic through coping strategies.

## Literature Review

### Organizational Resilience

When confronted with difficulties in life, certain people crack whereas some swing back (Ma et al., 2018). According to Bhamra et al. (2011), what is it that makes all the difference between people, societies, and countries surviving, adapting, and even prospering in the face of the most unforeseen life challenges? The solution is primarily based on resiliency qualities. Holling (1973, p. 17) invented the term “resilience” in his landmark book “Resilience and stability of ecological systems,” in which he claimed that resilience was a measure of the ability of a system to absorb changes while still surviving, and it determined how long it could last.

Resilience, as a holistic perspective, has been studied in a variety of areas. Walker et al. (2002) researched on ecological systems, Powley (2009) investigated positive psychology, Sheffi and Rice (2005) examined organizational management, and Hollnagel et al. (2006) analyzed engineering. According to Linnenluecke (2017), each field has its own definition of resilience; thus, operationalization and conceptualization differed among research. Werner and Smith (1977), for example, believed that resilience was critical for children’s development and growth, and therefore, several early studies focused on the individual resilience conceptualization for child development and growth. Management scholars and practitioners have recently been quite interested in conceptualizing and operationalizing organizational resilience (such as, Coutu, 2002; Weick et al., 2008; Oeij et al., 2017; Prayag et al., 2020) to investigate how organizations deal with difficulties and create new abilities and skills.

However, Burnard and Bhamra (2011) referred to organizational resilience as “the ability of an organization to generate awareness and reduce vulnerability to risky environments, to reinvent business strategies in the face of change, to continuously be aware of and adjust to changes, and to proactively react before the need for a change becomes obvious.” The concept of survival and recovery from adversity is entrenched in this definition, and it is the ability of an organization to adapt that is critical to its long-term viability (Salehi and Veitch, 2020). Firms should not just respond and adapt to various disruptions, but should also initiate, restore, and rebuild the structure of an organization and its affiliations to ensure that they can thrive in the adversity (Jiang et al., 2019). The literature recognizes two major components of organizational resilience: adaptive and planned (Filimonau et al., 2020; Prayag et al., 2020). Firm continuity and planning of risk management are examples of planned resilience, since they make use of current or established planning and resources. These are mostly activities that occur before a disaster. According to Chowdhury et al. (2019), adaptive resilience arises throughout the pandemic phase, as businesses create new skills by flexibly answering unexpected conditions. Adaptive resilience is built on risk knowledge, flexibility, and change preparedness of an organization (McCarthy et al., 2017).

According to Duchek (2020), resilience occurs at various levels within an organization, and these stages interrelate to define the resilience of the organization. The competence of an organization and procedures that are involved in resilience, according to Lengnick-Hall et al. (2011), are formed from a mixture of personal level characteristics such as, understanding, abilities, and skills. Thus, owners of small firms with high level of psychological capital (i.e., hope, optimism, resilience, and self-efficacy) are more probable to encourage the resilience of their firms.

### Psychological Capital

In organizational behavior theory, psychological capital has a long research history (Wu and Chen, 2018). According to Luthans et al. (2007), psychological capital has been advocated as a positive, distinctive, and long-lasting method that goes beyond typical human resource management and organizational behavior to recognize and develop the full potential of individuals. According to research in these fields (such as Baron et al., 2016; Ozturk and Karatepe, 2019; Sihag, 2020), psychological capital has been demonstrated to improve employee individual performance, as well as minimize employee absenteeism, stress, and attrition. Firm commitment and job satisfaction are positively associated with psychological capital, according to current research on the impact of psychological capital on individual performance, attitudes, and behaviors at work (Kang and Busser, 2018). Furthermore, according to Saud et al. (2021), psychological capital has a significant association with employee innovative and optimistic behaviors while dipping down negative attitudes of employees like anxiety. It can be considered that psychological capital can minimize employee anxiety and stress, and maximize employee productivity.

Psychological capital has been related to a variety of organizational behaviors and outcomes in the tourism literature. For instance, according to Schuckert et al. (2018), leadership styles in the hotel sector have a substantial influence on the psychological capital of an individual at work. Furthermore, psychological capital can have a substantial influence on the perceptions by hotel employees of their work-life quality and minimize the likelihood of turnover (Huang et al., 2020). Paek et al. (2015) emphasized that psychological capital improves job satisfaction and firm commitment, and lowers down the rate of stress. Positive innovative work behaviors can be influenced by psychological capital (Wang et al., 2021). On the other hand, these studies have not been undertaken in the context of small businesses in the tourism sector during the pandemic.

This study employs the definition by McFarlane and Norris (2006) of disaster, who defined it as “a potentially traumatic occurrence that is collectively experienced, has an immediate beginning, and is time-limited; disasters may be attributed to natural, technological, or human causes.” People who have been affected by the COVID-19 pandemic may feel extensive and long-term stress; thus, those who are affected by disasters must devise strategies for dealing with it (Hansel et al., 2020). Psychological capital, according to Prayag et al. (2020), plays a crucial role in the responses of people in stressful situations and can help to alleviate the stress and anxiety caused by disasters. It can also help people to increase their ability to take preventative measures (Pathak and Joshi, 2020). On the other hand, the recent studies concentrate on individual recovery in the event of a crisis rather than firm recovery through the role of an entrepreneur, which is a major gap in the literature on crisis restoration. When pressures are seen as demanding by an individual in the workplace, they can lead to positive organizational outcomes. However, negative organizational outcomes may grow if they are seen as dangerous (Prayag et al., 2020). The positive psychological capital of individuals can play a crucial role in their recovery from crises (Milosevic et al., 2017).

### Coping Strategies

The Transaction Model of Coping developed by Lazarus and Folkman (1984) describes the “process of a potentially stressful person-environment transaction.” Person and environment influencing variables, coping, cognitive evaluation, stress, and outcomes were all incorporated in their model. Moreover, according to Lazarus and Folkman (1987), cognitive evaluation and coping are critical determinants of traumatic person-environment interactions and their short and long-term effects. The transactional theory given by Lazarus and Folkman (1987) states that stress is a connection between the environmental expectations of a person and the availability of the resources to respond.

According to Lazarus and Folkman (1984), coping is a basic strategy to managing stress, and it refers to continuously changing cognitive and behavioral efforts to regulate a problematic person-environment connection. Furthermore, Lazarus and Folkman (1984) and, subsequently, Taylor and Schneider (1989) identified two categories of coping strategies: problem- and emotion-focused strategies. Lazarus and Folkman (1984) described both strategies as “problem-focused coping attempts to change the cause of stress or address issues by direct actions, while emotion-focused coping aims to avoid, distance, and selective attention to lessen or manage the emotional pain associated with stressors.” According to Folkman and Moskowitz (2004), both methods can be employed as an immediate stress response.

The recent study of Prayag et al. (2020) suggests that emotion-focused coping may be more effective in instances where an individual has less control over stresses. Some argue that determining the coping effectiveness requires examining the context or scenario (Zeidner, 1995). The literature on tourism has focused on coping strategies of tour guides in connection to stress and emotions while guiding, as well as how locals deal with the probable consequences of tourism growth (Min, 2014; Jordan et al., 2015). These studies, however, do not investigate it in the context of a pandemic. For instance, the study of Mason et al. (2010) on flood victims discovered that reasoning, detachment, and avoidance were the most common problem- and emotion-oriented coping strategies. Furthermore, as a result of the Canterbury earthquakes, Prayag and Orchiston (2016) identified that entrepreneurs employed coping strategies through a problem- and emotion-based approach to deal with gloomy tourism locations.

## Hypotheses Development

### Components of Psychological Capital

Positive psychology is the foundation of psychological capital and focuses on how people may maximize their abilities and skills by concentrating on the optimistic characteristics of their environments (Pathak and Joshi, 2020). Individuals who have accumulated psychological capital, according to Luthans (2002), are more adaptable to changes in the external as well as internal surroundings of an organization. Bandura (1997) defined self-efficacy as the belief of an individual in his/her capacity to do a task effectively and productively. The capability altitudes of individuals improve when they have faith in their own skills to complete a task. The ability of an individual to achieve desired goals and self-worth are just few of the components that lead to the higher level of reliance of an organization during a difficult environment (Prayag et al., 2020). Thus, individuals with greater level of self-efficacy will be more effective in terms of the resilience of an organization. Accordingly, this study proposes the following hypothesis:

H1a: Self-efficacy is a significant component of psychological capital to improve organizational resilience.

Hope, according to Snyder et al. (1991), is “a positive motivational state based on an interactively generated feeling of effective agency (goal-directed energy) and paths (planning to fulfill objectives).” Hope is having clear goals in mind and having a strategy in place to deal with any setbacks along the way to reach those goals (Pathak and Joshi, 2020). Hope can help individuals to manage their emotional discomfort while also motivating them to overcome problems (Prayag et al., 2020). Hope entails assessing the chances of achieving the objectives of an individual in the future. Therefore, hope is a way to attain organizational resilience (Othman and Nasurdin, 2011). The capability to transform goals and strive toward achieving goals in the face of hardship improves organizational resilience (Searle and Barbuto, 2011). Thus, this study proposes the following hypothesis:

H1b: Hope is a significant component of psychological capital to improve organizational resilience.

Extremera et al. (2009) discovered a relationship between optimism and resilience, which is defined as a general assumption of positive aspects that will occur in the future. Liu et al. (2012) explain that disasters provide a variety of obstacles for the tourism industry, and that managers must remain optimistic and minimize any changes from the strategy to achieve the intended objectives. Optimism theory, according to Scheier and Carver (1985), indicates that expectations about good outcomes lead to targeted action to attain the main objective. Furthermore, according to Luthans (2002), as a component of psychological capital, optimism is defined as having optimistic expectations for the present and the future. Higher goal orientation is associated with higher perceived success expectations (Carver et al., 2010; Pathak and Joshi, 2020). When confronted with a problem, optimistic individuals are better at regulating their negative emotions and are more adaptable and receptive to new experiences (Prayag et al., 2020). In the existing situation, once small tourism organizations are on the edge of shutting down their businesses, optimism might inspire owners/managers to consider new approaches to overcome a difficulty, enhancing their overall success. Thus, this study proposes the following hypothesis:

H1c: Optimism is a significant component of psychological capital to improve organizational resilience.

Luthans et al. (2007) explained that resilience is a combination of assets and resources inside an individual that helps them to gain confidence and see the positive aspect of a challenge. In the small tourism sector facing a variety of internal and external difficulties, such as changes in technologies and preferences of customer, climate change and natural disasters, and substantial arrangements during or after crises, owners/managers of hotels must have the ability to rebound in the occasion of any crisis (Pathak and Joshi, 2020; Prayag et al., 2020). According to Beasley et al. (2003), the capability to overcome difficulties during crisis and succeed in doing so gives an individual a sense of accomplishment that will create a sustainable impact on their business. Furthermore, the empirical study by Prayag et al. (2020) suggests that resilience correlates to better organizational resilience during times of crisis. Therefore, this study recommends the following hypothesis:

H1d: Resilience is a significant component of psychological capital to improve organizational resilience.

### Psychological Capital and Organizational Resilience

Psychological capital is concerned with how entrepreneurs may take advantage of their assets by focusing on the optimistic parts of their environment (Sarwar et al., 2017; Baluku et al., 2018). Entrepreneurs that have a high level of psychological capital are more adaptable to changes in the external environment (Tang, 2020). The study of Pathak and Joshi (2020) investigates the impact of psychological capital of hotel owners on organizational resilience during the COVID-19 pandemic. The study gathered data from 103 respondents in Indian urban cities, all of whom were small business owners of hotels, in order to better comprehend these interactions. The findings demonstrate that the psychological capital of small hotel owners can promote hope, self-efficiency, and optimism in order to realize circumstances and planning for future uncertainties. It also emphasizes how the association between psychological capital and organizational resilience has a substantial impact on the recovery process. Therefore, the following hypothesis is developed in this study:

H2: Psychological capital has a significant relationship with organizational resilience.

### Mediating Effect of Coping Strategies

The role of mediating variables in the relationship between stressful situations and adaptive outcomes has received a lot of attention lately (Boswell et al., 2004). In previous research (such as Folkman et al., 1986; Folkman and Lazarus, 1988; Parkes, 1994), coping has been shown to be a key mediating element in the person-environment connection. According to Folkman and Lazarus (1980), there are two forms of coping: “emotion-focused which aims to control or reduce the associated emotional discomfort, and problem-focused, which aims to change the problematic person-environment relationship.”

Anshel (2000) explains that coping has been defined in psychology as realistic and adaptable ideas, emotions, and relationships between a person and their environment in order to solve issues and handle stressful situations. Lazarus and Folkman (1984) established that coping encompasses “cognitive and behavioral efforts to handle particular external and internal pressures that are assessed as straining or surpassing a person’s resources.” The levels of coping of individuals are determined by the dynamics of change in their relationships with their surroundings (Drnovsek et al., 2010).

Psychological capital carries hope that not only allows entrepreneurs to cope with the emotional distress that comes with their circumstances, but it also motivates them to address the difficulties (Castro and Zermeño, 2020). Similarly, optimism is an essential element of psychological capital, since it is related to an emotion-focused coping strategy that allows an individual to reinterpret a circumstance in a more positive way (Liang and Cao, 2021). The third component of psychological capital is self-efficacy; it might be considered a problem-focused coping strategy, since it allows business owners to act in reaction to a situation (Prayag et al., 2020). Finally, according to Luthans (2002), the ability of individuals to cope successfully in the face of considerable change is defined as resilience, which encompasses prosocial behavior and issue-solving abilities. As a result, coping strategies, problem and emotion-focused, are involved with resilience.

Prayag et al. (2020) conducted a study to examine how psychological capital (optimism, hope, resilience, and self-efficacy) affects organizational resilience in a crisis. A qualitative research study in Kaikoura, New Zealand, found that participants were able to activate psychological capital to improve organizational resilience by adopting problem-focused and emotion-focused coping techniques. As small tourism firms manage the recovery process after a crisis, this may lead to better awareness of situation modifications in internal as well as external circumstances. Hence, the previous studies found a mediating effect of coping strategies (problem-focused and emotion-focused) between psychological capital and organizational resilience; therefore, this study hypothesized the following hypothesis:

H3a: Psychological capital has a significant relationship with problem-focused coping strategy.
H3b: Psychological capital has a significant relationship with emotion-focused coping strategy.
H4a: Problem-focused coping strategy has a significant relationship with organizational resilience.
H4b: Emotion-focused coping strategy has a significant relationship with organizational resilience.
H5: Problem-focused coping strategy has a mediating effect between psychological capital and organizational resilience.
H6: Emotion-focused coping strategy has a mediating effect between psychological capital and organizational resilience.

### Conceptual Framework

**FIGURE 1:**
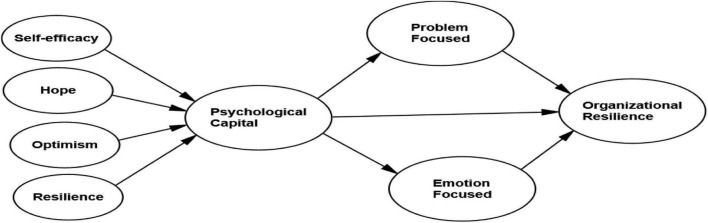
Conceptual framework.

As previously discussed, it is critical to comprehend psychological capital of small tourism firm owners/managers to build organizational resilience during a crisis. Therefore, the first aim of this study is to identify the significant influence of psychological capital on the resilience of tourism SMEs during the COVID-19 pandemic in Malaysia, and the second aim is to assess the mediating impact of coping strategies between psychological capital and the resilience of tourism SMEs during the COVID-19 pandemic in Malaysia. Hence, Figure 1 shows the conceptual model for this study, which conceptualized major components of psychological capital and their impact on organizational resilience. Moreover, the novelty of the study is to introduce the components of coping strategies (problem- and emotion-focused) as mediators to explore its effects between psychological capital and organizational resilience.

## Methodology

Ellen (1984) defines methodology as, “an articulated, theoretically informed approach to the production of data” (p. 9). Methodology refers to the study design, strategies, procedures, and approaches employed in a well-planned investigation to discover something new (Kivunja and Kuyini, 2017). Moreover, Crotty (1998) explained that methodology is the “strategy, plan of action, process or design” that notifies the selection of methods by researchers (p. 3). It assists the researcher in determining what kind of data is needed for a study and which collection of data methods/tools are best suited to their needs (Askarzai and Unhelkar, 2017). It is concerned with how scholars learn about the domain or a particular component of it.

Venkatesh et al. (2016) argued that the methods used to lead the research must be on the same path as the main objective. Hence, a quantitative method was used in this study to test the hypotheses and to achieve the aim of the study. According to Queirós et al. (2017), quantitative approaches are structured method for uniting inferential reason with defined empirical investigations in order to find and verify a set of probabilistic causal relationships. Similarly, Smith and Hasan (2020) use a quantitative approach to assist researchers in establishing statistical proof of links between dependent and independent constructs.

Data collection approaches include self-administered surveys, telephone interviews, and personal interviews (Kraus and Augustin, 2001). This study used a self-administered questionnaire known as a “drop-off survey.” Using this approach, the researcher or a representative of the researcher (in this case, enumerators) should travel to the respondent’s location and hand out questionnaires (Chen et al., 2003).

Data were collected from small tourism firm owners in different cities of Malaysia, such as Kuala Lumpur, Malacca, Kota Kinabalu, Petaling Jaya, Kuching, George Town, and Ipoh. A total of 800 survey questionnaires were sent to SMEs Crop Malaysia-registered small tourism enterprises. The data were acquired using a stratified random sample method with 82.5% response rate. A cover letter outlined the objective of the study, as well as the criteria and directions for completing the questionnaire. Hence, only 644 questionnaires were completed in all respects after the data were screened and then imported for further assessment.

Items were chosen for this study to assess the effect of psychological capital on small tourism organizational resilience and the mediating influence of coping strategies. To measure the variables of the study, the items were adapted from the literature. The items for measuring psychological capital (hope, optimism, self-efficacy, and resilience) were adapted from Luthans et al. (2007) and Pathak and Joshi (2020). To measure the mediating effect of coping strategies (problem-focused and emotion-focused), items were adapted from Scherer et al. (1988) and Minnie et al. (2015). Items from Orchiston and Higham (2016) and Pathak and Joshi (2020) were adapted to measure organizational resilience.

To evaluate the hypotheses, structural equation modeling (SEM) was performed on the data in a two-stage approach to analyze the structural and measurement models using AMOS 21.0. The researchers performed SEM to examine the fundamental associations between the constructs in this study. In the first step, a confirmatory factor analysis (CFA) was performed to assess the convergent validity of the measurement model and causal relationship among adapted items and variables (Hair et al., 2014; Kline, 2015). The structural model was used in the second stage to examine the relationship between the exogenous variable (psychological capital) and the endogenous variables (coping strategies and organizational resilience).

## Results

### Demographic Characteristics

The data were collected from 644 owners/managers of small enterprises in the tourism sector of various cities in Malaysia. Set (2013) identified tourism business activities, which was followed by this study. The economic activities of the respondents were: accommodation services; transportation services; art, entertainment and recreation services; food and beverage services; miscellaneous tourism services; travel agency; tour operator; and tourism guide services. Furthermore, gender, age, marital status, education, religion, and income level of the respondents have all been categorized. A demographic analysis was performed based on the items in the questionnaire to establish the backgrounds of the respondents, as shown in Table 1.

**TABLE 1:** Profile of respondents.

Variables		Number	Percentage
Gender	Male	409	63.5%
	Female	235	36.5%
Age	Less than 25	53	8.2%
	26–35	87	13.5%
	36–45	122	18.9%
	46–55	279	43.4%
	Above 55	103	16.0%
Marital status	Single	86	13.4%
	Married	491	76.2%
	Widow	18	2.8%
	Divorced	49	7.6%
Education	High school or less	91	14.1%
	Diploma	231	35.9%
	Bachelor degree	204	31.7%
	Master	105	16.3%
	Doctorate	13	2.0%
Religion	Muslim	203	31.5%
	Hindu	69	10.7%
	Christian	225	35.0%
	Buddhist	136	21.1%
	Others	11	1.7%
Income level	Less than RM 4,000	87	13.5%
	4,001–5 K	98	15.2%
	5,001–6 K	107	16.6%
	6,001–7 K	196	30.5%
	7,001 K or above	156	24.2%
Business activities	Accommodation services	311	48.2%
	Transportation services	93	14.5%
	Art, entertainment and recreation services	28	4.3%
	Food and beverage service	74	11.5%
	Miscellaneous tourism services	108	16.8%
	Travel agency, tour operator, and tourism guide services	30	4.7%

### Normality Statistics

Testing for multivariate normality is the most important assumption in structural equation modeling (SEM). SEM assumes continuous variables in the research and produces the best results (Andreassen et al., 2006). Skewness and kurtosis values in the ± 3 range, according to Ghasemi and Zahediasl (2012), may indicate that a variable is distributed normally. In this study, the statistical value of skewness and kurtosis for each construct is determined and reported in Table 2.

**TABLE 2:** Descriptive statistics.

Constructs	Range	Mean	Std. Dev.	Skewness	Kurtosis
Self-efficacy	1–7	4.28	0.47	0.087	–0.504
Hope	1–7	5.34	0.52	–1.022	–0.714
Optimism	1–7	4.87	0.48	0.136	–1.603
Psychological resilience	1–7	5.15	0.38	1.217	0.026
Problem-focused	1–7	5.03	0.61	–0.170	–0.365
Emotion-focused	1–7	4.76	0.42	0.144	0.096
Organizational resilience	1–7	4.93	0.51	0.107	0.366

### Reliability

According to Tarhini et al. (2016), “internal consistency signifies the extent to which respondents are reliable across the items mentioned in the questionnaire as a measurement scale.” Furthermore, Pallant (2020) explained that Cronbach’s alpha of more than 0.7 is considered as good internal consistency. In this study, Cronbach’s alpha was used to measure the internal consistency for the following variables: self-efficacy (α = 0.884), hope (α = 0.895), optimism (α = 0.843), psychological resilience (α = 0.869), problem-focused (α = 0.858), emotion-focused (α = 0.891), and organizational resilience (α = 0.838). According to Kline (2015), variables that have reliability of more than 0.8 is considered as very good or excellent internal consistency.

### Discriminant Validity

Discriminant validity refers to the degree to which one construct differs from another (Cheah et al., 2018). According to Voorhees et al. (2016), the correlation between the two conceptions should be lower than 0.85. In this study, the discriminant validity of all the constructs was evaluated using SPSS statistics version 22.0. The results are presented in Table 3, and show that the correlation among the constructs is less than 0.85.

**TABLE 3:** Correlation of the constructs.

	1	2	3	4	5	6	7
Self-efficacy	1						
Hope	0.33	1					
Optimism	0.39	0.28	1				
Psychological resilience	0.27	0.31	0.38	1			
Problem-focused	0.39	0.33	0.44	0.36	1		
Emotion-focused	0.47	0.41	0.26	0.34	0.42	1	
Organizational resilience	0.45	0.27	0.38	0.32	0.29	0.32	1

### Assessment of Confirmatory Factor Analysis

Confirmatory factor analysis (CFA), the first step of two-stage SEM statistical method, allows a researcher to rectify measurement error during the assessment of multiple variable relationships (Ong and Puteh, 2017). Maximum likelihood valuation is performed to estimate the associations among the variables and their corresponding indicator items. Shek and Yu (2014) identified that in CFA the factor loading of each item should be 0.6 or above to be considered acceptable. According to the findings, the factor loading of items and fit indices are in the acceptable range; summarized CFA results are shown in Table 4.

**TABLE 4:** Results after CFA.

Constructs	Chi-square	CMIN/df	GF1	AGFI	CFI	RMESA
Self-efficacy	19.723	2.554	0.968	0.934	0.979	0.071
Hope	20.108	2.187	0.972	0.939	0.983	0.074
Optimism	18.629	2.493	0.961	0.928	0.973	0.073
Psychological resilience	21.245	2.292	0.971	0.929	0.986	0.067
Problem-focused	20.710	2.361	0.965	0.933	0.978	0.072
Emotion-focused	19.961	2.187	0.958	0.924	0.984	0.077
Organizational resilience	21.878	2.388	0.963	0.932	0.974	0.075

### Assessment of Overall Measurement Model

**FIGURE 2:**
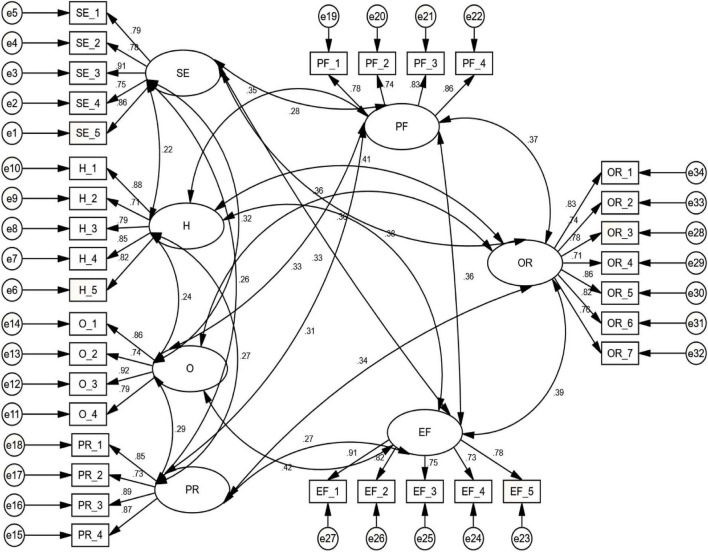
Measurement model.

Following the CFA validation findings, the overall measurement model was assessed. The model that links the latent constructs to their indicators is referred to as the overall measurement model (Kline, 2015). The findings of this study demonstrated that the goodness-of-fit indices for the overall measurement model was well-fitted, such as RMSEA of 0.043 and chi square value of 623.433 with 643 degrees of freedom, GFI = 0.916, AGFI = 0.919, CFI = 0.934, and CMIN/df = 1.546. The overall measurement model is depicted in Figure 2.

### Average Variance Extracted and Composite Reliability

The findings of the study show that the composite reliability (CR) values of all the constructs are over 0.7, indicating that the variables are internally consistent. Furthermore, the value of average variance extracted (AVE) for all the variables was found to be greater than 0.5, indicating that the constructs are convergent valid (Fornell and Larcker, 1981).

As described by Fornell and Larcker (1981), “the discriminant validity was determined by comparing the square root of each AVE in the diagonal with the correlation coefficients (off-diagonal) for each construct in the relevant rows and columns.” The statistical results of the average variance extracted (AVE) value in this study is more than 0.6 for all the constructs. Table 5 shows the AVE and CR findings.

**TABLE 5:** Convergent validity evaluation.

Items	Measurement path	Factor loading	CR	AVE
**Self-efficacy**				
SE_1	During the COVID-19 pandemic, I am confident in assessing a long-term problem and finding a solution	0.79	0.911	0.673
SE_2	During COVID-19, I am confident in expressing my plans in online meetings with management	0.78		
SE_3	I’m confident in my ability to contribute to conversations concerning the company’s COVID-19 strategy	0.91		
SE_4	During the COVID-19 pandemic, I am confident in my ability to assist in the setting of targets and goals	0.75		
SE_5	During the pandemic of COVID-19 I’m comfortable approaching people outside the organization to address issues	0.86		
**Hope**				
H_1	During the covid-19 pandemic, I am working hard to achieve my professional objectives	0.88	0.906	0.660
H_2	Any disaster or crisis can be avoided in a variety of ways	0.71		
H_3	I view myself as being rather effective at work during Covid-19	0.79		
H_4	During any crisis or disaster, I can think of a variety of strategies to achieve my current company goals	0.85		
H_5	Despite the pandemic, I am achieving the company goals I set for myself	0.82		
**Optimism**				
O_1	When things at business are uncertain for me, I typically hope for the best for the company	0.86	0.898	0.689
O_2	Even in the middle of the covid-19 pandemic, I try to see the positive side of things when it comes to my business	0.74		
O_3	In terms of work, I’m optimistic about what will happen to my company after Covid-19	0.92		
O_4	I’m approaching this pandemic with the mindset that “every cloud has a silver lining.”	0.79		
**Psychological resilience**				
PR_1	At business, I generally deal with crises/disasters in one way or another	0.85	0.903	0.701
PR_2	If I have to, I can work “on my own,” as it were	0.73		
PR_3	I generally take difficult situations at business, like as the covid-19 pandemic, in stride	0.89		
PR_4	As a hotel owner/manager, I believe I have the ability to do things	0.87		
**Problem-focused**				
PF_1	Make use of my experience; I’ve been in a similar scenario previously	0.78	0.879	0.646
PF_2	Come up with a few different ways to solve the problem	0.74		
PF_3	I attempt to investigate the situation in order to have a better understanding of it	0.83		
PF_4	I’m developing a strategy and sticking to it	0.86		
**Emotion-focused**				
EF_1	When I thought about current crisis or was reminded of it, I tried not to become upset	0.91	0.899	0.641
EF_2	I avoid anyone or anything that reminds me of current crisis	0.82		
EF_3	I had a slew of intense emotions about current situation	0.75		
EF_4	I am trying to concentrate on solution of current situation	0.73		
EF_5	I attempted to forget about current crisis and make a plan for future recoveries	0.78		
**Organizational resilience**				
OR_1	Our hotel’s goals for what’s vital during and after COVID-19 are well-defined	0.83	0.919	0.701
OR_2	Our hotel is forming relationships with groups in which we may be required to collaborate during and after COVID-19	0.74		
OR_3	During disasters like COVID-19, our hotel has the resources to absorb some unforeseen adjustment	0.78		
OR_4	Our hotel has made it a priority to be prepared to respond to unforeseen disasters such as COVID-19	0.71		
OR_5	Our hotel’s approach to preparing for the unexpected is suitable	0.86		
OR_6	We are recognized as a hotel for our ability to use information in new ways	0.82		
OR_7	Our hotel is capable of making quick decisions	0.76		

### Assessment of Structural Model

**FIGURE 3:**
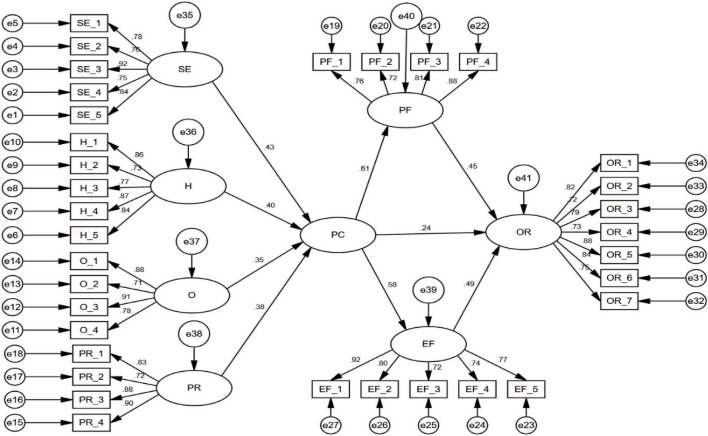
Structural model.

The structural model (stage 2) was used to investigate the relationship of psychological capital and organizational resilience. AMOS 21.0 was used to evaluate the data. In contrast to earlier studies (such as Hair et al., 2014; Kline, 2015), the goodness-of-fit indices are tested in this study, as shown in Figure 3. The results were well-fitted, with an RMSEA of 0.037 and a chi square value of 565.886 with 643 degrees of freedom, GFI = 0.915, AGFI = 0.909, CFI = 0.941, and CMIN/df = 1.674.

Table 6 shows the results of direct relationships. The study used a significance level of 1.96 for the *z*-value and a *p*-value < 0.05, as recommended by Kline (2015). Hypotheses H1a, H1b, H1c, H1d, H2, H3a, H3b, H4a, and H4b were statistically significant in the evaluation of hypothesized relationship.

**TABLE 6:** Testing direct relationship.

Paths	ß	*Z*-value	*P*-value	Significant
H1a: SE– > PsyCap	0.43	5.362	***	Yes
H1b: H– > PsyCap	0.40	5.038	***	Yes
H1c: O– > PsyCap	0.35	4.429	***	Yes
H1d: PR– > PsyCap	0.38	4.767	***	Yes
H2: PsyCap– > OR	0.24	3.398	0.003	Yes
H3a: PsyCap– > PF	0.61	6.924	***	Yes
H3b: PsyCap– > EF	0.58	6.673	***	Yes
H4a: PE– > OR	0.45	5.891	0.007	Yes
H4b: EF– > OR	0.49	6.189	***	Yes

***p ≤ 0.001.

### Assessment of Mediating Effect

Relationships that proposed the mediating influence of problem-focused and emotion-focused coping strategies on psychological capital and organizational resilience were investigated in this study using H5 and H6. The indirect effect of a problem-focused coping strategy was 0.27 (0.61 × 0.45 = 0.27), whereas the direct effect of psychological capital on organizational resilience was 0.24. Similarly, the indirect effect of an emotion-focused coping strategy was 0.28 (0.58 × 0.49 = 0.28), whereas the direct effect was 0.24. The form of mediation used here is partial mediation, because the direct effect remains significant after the mediator enters the model (Awang et al., 2015). As a result, it was discovered that both problem-focused and emotion-focused coping techniques played a role in mediating the relationship.

We implement the bootstrapping method to validate the mediation analysis after confirming direct and indirect effects. According to Awang et al. (2015), the researchers calculated the standardized indirect and direct effects, as well as their significance levels, using a 1,000-bootstrap sample with a bias adjustment of 95%. Hypothesis H5 and H6 were accepted. The statistical findings of the investigation are shown in Table 7.

**TABLE 7:** Assessment of mediating effect (bootstrapping results).

Constructs	Effect	Significant
Problem-focused	0.27 (Indirect effect)	Yes
	0.24 (Direct effect)	
Emotion-focused	0.28(Indirect effect)	Yes
	0.24 (Direct effect)	

## Discussion

There has been no research on Malaysian small tourism that investigated the relationship between psychological capital and organizational resilience. Previous research (such as Biggs et al., 2012; Orchiston, 2013; Dahles and Susilowati, 2015; Kang et al., 2018) has been carried out in countries other than Malaysia to explore the organizational resilience in tourism organizations, as well as the function of psychological capital in large firms. This research explored the influence of the psychological capital of small business owners/managers on organizational resilience, and the novelty of the study is to identify the mediating effect of coping strategies between psychological capital and organizational resilience. According to the findings of the study, psychological capital (hope, self-efficacy, resilience, and optimism) effectively enhanced small tourism firm resilience (Pathak and Joshi, 2020; Prayag et al., 2020). Furthermore, the findings suggest that both problem-focused and emotion-focused coping strategies partially mediate the relationship between psychological capital and organizational resilience. Therefore, the findings of the study were compatible with the research hypotheses and able to answer the research questions.

The first research question (RQ1) was: *“Does psychological capital influence the resilience of tourism SMEs during Covid-19 pandemic in Malaysia”?*

Research question 1 was related to the test path of self-efficacy. Hope, optimism, and resilience are predictors of psychological capital during the COVID-19 pandemic. Hypothesis H1a was found to predict self-efficacy as a positive component of psychological capital (path 0.43, z-score 5.362, and *p*-value 0.05). Hypothesis H1b found that hope is a predictor of positive psychological capital, with a path of 0.40, a z-score of 5.038, and a *p*-value <0.005. Hypothesis H1c, optimism to the psychological capital path, was tested; hence, coefficient 0.35, z-score 4.429, and *p*-value < 0.05 validate the hypothesis. Hypothesis H1d, resilience to the positive psychological capital path, was assessed. Path coefficient 0.38, z-score 4.676, and *p*-value < 0.05 confirms that resilience predicts positive psychological capital. H1a–H1d testify that positive psychological capital comprised the four predictors named above according to the theory of Luthans (2002). Hence, the obtained results are according to the theory. However, it is important to note that the authors used the COVID-19 phenomenon to measure the contribution of positive psychological capital in this study. A more suitable and efficient technique of human psychology should be applied to human resources and psychological capital (PsyCap). It aspires to save the resources of an organization and use them to solve problems and depreciation. Managers are first-line leaders who actively participate in crisis management. Using positive psychological capital, such as hope, self-efficacy, resilience, and optimism, they can make flourish tourism-related business value and prestige. According to Luthans et al. (2007), positive psychological development happens in humans by their self-efficacy. Individuals with high positive psychological capital can make positive attributions utilizing optimism to achieve goals now and in the future. This also refers to achieving goals and people may perceive a bright future ahead of them. Finally, when faced with obstacles and hardship, resiliency operates in the human mind to achieve a goal.

The second research question (RQ2) is: *“Does coping strategies mediate the relationship between psychological capital and the resilience of tourism SMEs during Covid-19 pandemic in Malaysia”?*

H2 determined that positive psychological capital is a predictor of organizational resilience. Hence, path PsyCap to OR 0.24, z-score 3.398, *p*-value < 0.05 confirm the positive and significant relationship between psychological capital and organizational resilience. The results of the study are in agreement with the previous findings of Pathak and Joshi (2020) and Prayag et al. (2020), who, among others, found that a small tourism owner/manager psychological capital might encourage hope, self-efficiency, resilience, and optimism in order to realize situations and resilience plan for uncertainty. Thus, the finding contributes to the tourism literature that focuses on how a small tourism business owner/manager employs psychological capital to activate organizational resilience that benefits their firm.

H3a was formulated to test the relationship between PsyCap and problem-focused strategy. Coefficient 0.61, z-score 0.692, and *p*-value < 0.05 confirm the positive and significant relationship between psychological capital and problem-focused strategy. H3b hypothesized that positive psychological capital is required for emotionally focused strategy during the COVID-19 pandemic. Coefficient 0.58, z-score 6.673, and *p*-value < 0.05 confirm the positive and significant relationship. It is pertinent that positive psychological capital predicts an emotionally focused strategy. Positive psychological capital brings emotional consistency in humans. H4a determined that a problem-focused strategy builds organizational resilience. Path coefficient 0.45, z-score 5.891, and *p*-value 0.05 confirm the positive and consistent relationship. H4b determined that emotionally focused strategy builds organizational reliance; hence obtained coefficient 0.49, z-score 6.189, and *p*-value 0.05 confirm the positive and significant relationship. It is evident in path testing that individuals feel more contented and emotionally focused than problem-focused.

According to Folkman and Lazarus (1988), coping has been proven to be a major mediating factor in the person-environment relationship. Moreover, they identified that problem- and emotion-focused coping are two key components of coping strategies. Therefore, this study investigated the mediating effect of problem- and emotion-focused coping strategies between the psychological capital of small business owners and organizational resilience in the tourism sector during the COVID-19 pandemic, and a partial mediating effect was confirmed by the findings. The study implies that small business owners/managers employ various coping strategies to trigger business recovery during the pandemic. The results extend to the research of Prayag et al. (2020) by demonstrating that, in addition to psychological capital, multiple coping strategies have an indirect impact on organizational resilience during a crisis.

## Conclusion

This study focuses on the impact of psychological capital on organizational resilience and the mediating role of coping strategies in helping small tourism business owners/managers to develop long-term resilience. The findings show that psychological capital plays an important role in enhancing coping strategies, and that coping plays a vital role for owners/managers in the rehabilitation of small businesses during or after a crisis, implying that the psychological capital of owners/managers is critical in building organizational resilience.

The rising literature on the tourism sector (such as Pathak and Joshi, 2020; Prayag et al., 2020) is paying attention to owners/managers of large tourism organizations to adapt to changes and endure disasters. On the other hand, small tourism businesses have not paid attention to it as compared to the large tourism businesses, and there is lack of disaster preparedness among them. In the context of small firms in the tourism sector, organizational resilience is more dependent on the capability of the owner/manager to analyze the situation, and adoption of innovation to overcome disturbances (Orchiston and Higham, 2016). Constructing resilience in small firms in the tourism sector needs additional attention to the psychological capital of owners or managers as compared to infrastructural improvements. The psychological capital of owners and managers of small tourism firms will assist them in developing coping strategies and resilience to counter the difficulties created by COVID-19.

## Implications of the Study

From a theoretical perspective, this research contributed to the existing literature in the domains of tourism, entrepreneurship, and disaster management. Previous empirical research has focused on large tourism firms in either psychological capital (Ozturk and Karatepe, 2019; Mao et al., 2020) or organizational resilience (Pathak and Joshi, 2020; Sobaih et al., 2021), but none has examined how coping mechanisms connect psychological capital and organizational resilience in small tourism enterprises. Therefore, this study shows empirically how entrepreneurs may employ problem- and emotion-focused coping mechanisms stimulated by psychological capital to minimize the impacts of the COVID-19 crisis on small tourism firms. According to this research, the psychological capital of owners/managers of small tourism firms supports the strengthening of organizational resilience to quickly recover and survive from disaster-related difficulties. This is one of the reasons why most tourism organizations chanting are a new slogan of sustainability, most critical is to improve organizational resilience strategies, and this study contributes to this goal by emphasizing the importance of the psychological capital of owners and the use of coping strategies to enhance organizational resilience.

From a practitioner perspective, the findings of this study have clarified the importance of psychological capital for the resilience of small firms in the tourism sector. Small tourism firm owners/managers appear to have made their firms develop resilience through psychological capital and coping techniques used by successful peer mentors or role models during the COVID-19 pandemic. Therefore, providing small firms in the tourism sector the chance to be led by industry experts who have successfully employed psychological capital and coping strategies in their businesses the during pandemic would be an effective way to aid business recovery. According to Prayag et al. (2020), small company owners/managers can master the stepping method, which involves breaking down difficult and long-term goals into smaller and more achievable milestones. From a management standpoint, the study has identified the necessity for small tourism firms to recognize the value of personal assets, such as the psychological capital of owners, and to develop them in an effort to combat the problems created by COVID-19. Psychological capital may be used to develop a strong social capital that will assist in discovering approaches to achieve organizational goals and increase the overall resilience. The psychological capital of owners/managers may restore faith in the capacity of an organization to endure difficulties and accomplish intended objectives.

## Limitations and Future Scope of the Study

First, the cross-sectional approach utilized in this study has limitations when it comes to understanding the causal relationships among psychological capital, organizational resilience, and coping strategies. Longitudinal studies should be conducted in the future to investigate certain causal relationships. Second, this study was confined to major cities in Malaysia, owing to severe COVID-19 sanctions and closure. Nevertheless, the research was not limited to small tourism businesses in cities, as it also covered rural areas. Researchers should do research on rural tourism businesses or comparatively examine the organizational resilience of rural and urban small businesses in the tourism sector. Third, this study focused on the impact of psychological capital in owner/managers of small tourism businesses, but there was no assessment of psychological capital in workers. Multi-level assessment and diversification of information sources should be considered in a future study.

## Data Availability Statement

The original contributions presented in the study are included in the article/supplementary material, further inquiries can be directed to the corresponding author/s.

## Ethics Statement

Ethical review and approval was not required for the study on human participants in accordance with the local legislation and institutional requirements. The patients/participants provided their written informed consent to participate in this study.

## Author Contributions

All authors listed have made a substantial, direct, and intellectual contribution to the work, and approved it for publication.

## Conflict of Interest

The authors declare that the research was conducted in the absence of any commercial or financial relationships that could be construed as a potential conflict of interest.

## Publisher’s Note

All claims expressed in this article are solely those of the authors and do not necessarily represent those of their affiliated organizations, or those of the publisher, the editors and the reviewers. Any product that may be evaluated in this article, or claim that may be made by its manufacturer, is not guaranteed or endorsed by the publisher.
